# Effects of *Aeromonas veronii* and Its Vaccine on Immune-Related Gene, Liver Transcriptomics, and Gill Microbiota in Crucian Carp

**DOI:** 10.3390/vaccines14040307

**Published:** 2026-03-29

**Authors:** Junbo Wang, Shiyong Huang, Yingtiao Lai, Ping Wang, Feifei Wang, Dahui Pan, Fei Zhao, Hua Gong

**Affiliations:** 1 Pisa Marine Graduate School, Zhejiang Ocean University, Zhoushan 316000, China; wjb011127@163.com (J.W.);; 2 Key Laboratory of Aquatic Animal Immune Technology of Guangdong Province, Key Laboratory of Fishery Drug Development, Ministry of Agriculture and Rural Affairs, Pearl River Fisheries Research Institute of Chinese Academy of Fishery Sciences, Guangzhou 510380, China; 3 Guangzhou Jianghe Bio-Technology Co., Ltd., Foshan 528000, China

**Keywords:** *Aeromonas veronii*, crucian carp, vaccination, challenge, mechanism, immune-metabolism, gill symbiotic microbiota

## Abstract

**Background**: *Aeromonas veronii* is an important bacterial pathogen in crucian carp and can cause serious disease outbreaks and substantial economic losses in aquaculture. **Objectives**: To evaluate how *A. veronii* infection and its inactivated vaccine modulate immune responses in *Carassius auratus*. **Methods**: 270 juveniles were allocated into three groups: a saline-injected control group (Ctrl), a vaccination group receiving an inactivated *A. veronii* vaccine (Vac), and an artificial infection group (AIG) subjected to stimulation. Liver, spleen, head kidney, gill, and intestine samples were collected from fish after anesthesia. The relative transcript levels of *IgM*, *IgD*, *BAFF*, *MHCII*, *CD4*, *BCL6*, *MyD88*, and *NF-κB* were quantified. For liver transcriptome analysis, the effective library concentration was determined. And the 16S rRNA gene resulting reads of fish gill symbiotic microbiota were processed for downstream bioinformatic analysis. **Results**: The results showed that the Vac achieved an RPS of 73.33%, and vaccination significantly upregulated multiple immune-related genes in different fish organs. With *BAFF* transcription across organs emerging as a robust sentinel readout. The Pearson correlation coefficient (*r*) of BAFF between other genes were all ≥0.8. GO and KEGG enrichment analyses indicated that AIG had more DEGs than Vac (5885 vs. 4008) and Ctrl (6910 vs. 6178), respectively. Some genes in AIG revealed significant over-representation of immune pathways, such as *BCL6*, *MyD88*, and *NF-κB*. The fish gill microbiota comprised a diverse set of low-abundance taxa, the phylum level was dominated by Proteobacteria and Fusobacteriota across all groups; whereas, the Vac group remained broadly closer to the Ctrl group in overall composition. **Conclusions**: These results indicated marked post-challenge immune–metabolic coupling in the liver, and suggested coordinated immunophysiological interplay between the liver and the spleen. Gill microecology of symbiotic bacteria was affected by vaccination or challenge reactions, which in turn affects the health of the gills or the organism itself.

## 1. Introduction

Crucian carp (*Carassius auratus*) is one of the most important farmed species in China, and its diseases are often reported to be caused by *Aeromonas veronii* [1,2,3]. As a ubiquitous inhabitant Gram-negative pathogen of aquatic environments, *A. veronii* infects a variety of freshwater fish species resulting in heavy economic losses for aquaculture, which can manifest serious diseases such as hemorrhagic septicemia, dropsy, ulceration, asymptomatic septicemia and related clinical signs [2,3]. Vaccination is the most environmentally friendly disease control strategy and one of the most effective methods of combating threatening diseases in fish [4].

Several immune-related molecular indexes are widely used to characterize immunophysiological differences among tissues and to delineate host responses. Pattern recognition through Toll-like receptor (*TLR*) constitutes a principal gateway signal for bacterial early infection [5]. In fish, rapid mucosal sensing of pathogens and immune stimuli relies heavily on the conserved *TLR–MyD88–NF-κB* axis, which is commonly employed as a proxy module to gauge the magnitude of vaccine-elicited activation and challenge-induced responses [6]. *MyD88* acts as a key adaptor downstream of most TLR and drives the activation of transcription factors such as *NF-κB*, thereby enabling a prompt response to microbial invasion [4,7]. In teleosts, *MHCII*-associated factors and *CD4*-associated factors have been demonstrated to participate in both mucosa-associated lymph tissues (MALT) and systemic immune organs, contributing to infection control and immune regulation [8,9]. Among humoral components, *IgM* is one of the predominant systemic antibody isotypes in teleosts. *IgD* has been linked to specific B-cell subsets and MALT, thus it is informative for describing localized, tissue-level B-cell responsiveness [10]. *BAFF* (B-cell activating factor) is a key cytokine that supports B-cell survival, maturation, and antibody production, so likewise contributes to B-cell homeostasis in fish [11]. *BCL6*, a transcription factor involved in shaping B-cell differentiation programs, is closely associated with the quality and trajectory of antibody responses [12,13]. Consequently, evaluating *BAFF/BCL6* together with *IgM/IgD* facilitates an integrated interpretation of immune modulation across three complementary layers: B-cell maintenance, differentiation programming, and effector output.

Regarding continuous exposure to microbe-rich aquatic environments, fish MALT in organs such as gill and intestine constitute the first line of defense against pathogen entry [2,4]. The gills of fish serve as organs for gas exchange and are also one of the mucosal interfaces most prone to contact and colonization by pathogens. Therefore, the effectiveness of the immune defense of the gill MALT not only depends on the innate and adaptive immunity of the host itself but also highly relies on the “ecological barrier” formed by the microbial community on the mucosal surface. Adaptive immunity may regulate the anti-inflammatory pathway to cause inflammatory immune attack or tolerance, and adjust the distribution of intestinal symbiotic microbiota. These studies have discussed the unidirectional changes in immune factors and symbiotic bacteria, but there is no comprehensive evaluation of their synergistic effects.

In this study, crucian carp were intraperitoneally, artificially infected with an *A. veronii* suspension to reproduce an acute bacterial disease course. The resulting immune transcriptional profiles were then compared with those observed following vaccination, focusing on the immune-related genes described above. The impact of different immune treatments on the symbiotic microbiota and the influence of the symbiotic microbiota on the organism are discussed. This design aimed to delineate the systemic response signatures associated with vaccination versus pathogen challenge, thereby providing a molecular framework to support subsequent vaccine efficacy evaluation and mechanistic optimization.

## 2. Materials and Methods

### 2.1. Materials

Juvenile crucian carp (10 ± 2 g) were obtained from Foshan Sanshui Baijin Aquatic Seed Co., Ltd. (Foshan, China), and acclimated in glass tanks at the experimental fish rearing facility of the Pearl River Fisheries Research Institute, CAFS. After a 5-day acclimation period, fish were enrolled in the experiment. During the trial, fish were fed twice daily (09:00 and 17:00) at 3–4% of body weight. Each morning, half of the tank water volume was renewed and fecal wastes were removed. Fish behavior and health status were monitored daily, dead individuals were promptly removed and recorded.

The *A. veronii* strain AVCA07 was maintained in our laboratory and prepared by Guangdong Jianghe Biotechnology Co., Ltd. (Foshan, China). The bacterium was first streaked onto nutrient agar and incubated at 28 °C for 24 h. Then, a single colony was inoculated into Luria-Bertani (LB) broth and cultured at 28 °C with shaking at 200 rpm (THZ-98C, Shanghai Yiheng Scientific Instruments Co., Ltd., Shanghai, China) for 18 h. Upon completion, cultures were sampled to confirm a viable count of ≥5 × 10^9^ CFU/mL for subsequent use.

Formaldehyde solution was added with 0.30% final concentration of the *A. veronii* suspension as inactivating at 28 °C for 48 h, with gentle mixing performed every 4 h to ensure uniform inactivation. Two tubes of nutrient broth medium were inoculated in the same manner and incubated at 28 °C for 5 days; both should show no microbial growth. Then, the inactivated cells were harvested by centrifugation at 8000 rpm for 15 min, portioned and refrigerated after passing the sterility test.

### 2.2. Experimental Grouping

270 juvenile crucian carp were allocated randomly into three treatments, each with three replicates (30 fish per replicate). Two experimental groups were established: the Vac group (Vac), which was intraperitoneally injected with 0.2 mL (≥5 × 10^9^ CFU/mL) of the inactivated *A. veronii* vaccine, and the artificial infection group (AIG), which was intraperitoneally injected with 0.2 mL of a virulent *A. veronii* suspension (viable count ≈ 6.5 × 10^6^ CFU/mL). While the Ctrl group (Ctrl) received an intraperitoneal injection of 0.2 mL 0.85% physiological saline.

### 2.3. Sample Collection

Fish were fasted for 24 h prior to sampling, during which the water was not renewed. At 8 h post-injection (8 hpi), ten fish were randomly selected from the surviving individuals in each group. The fish were anesthetized with MS-222 (Merck Life Science (China) Co., Ltd., Wuxi, China) and then dissected. The head kidney, spleen, liver, intestine, and gill tissues were collected and immediately stored at −80 °C (HD-86L390, Hisense Ronshen, Guangzhou, China) until further analyses. In addition, liver samples were collected for transcriptome sequencing analysis. For the challenge test, 15 fish per group were subjected to infection by intraperitoneal injection of a suspension of *A. veronii*. Then, these fish were monitored for 5 days, and daily mortalities were recorded. Mortality was calculated based on disease-specific deaths.

### 2.4. Quantification of Immune-Related Gene Expression (qPCR)

Total RNA was extracted from the collected tissues according to the manufacturer’s instructions (Tiangen Biotech, Beijing, China). RNA purity (1.8 < A260/A280 < 2.0) and concentration were determined using a NanoDrop 2000 spectrophotometer (Thermo Fisher Scientific, Waltham, MA, USA). RNA integrity was assessed by 1.5% agarose gel electrophoresis and verified with an Agilent 2100 Bioanalyzer system (Agilent Technologies, Santa Clara, CA, USA). Purified RNA was subsequently reverse-transcribed into cDNA using a Perfect Real Time kit (TaKaRa, Otsu City, Japan). And Gene-specific primers were designed based on crucian carp sequences available in NCBI GenBank using Primer Premier 5.0; primer information is provided in Table 1. Quantitative real-time PCR (qPCR) was performed using TB Green^®^ Premix Ex Taq™ II (TaKaRa, Japan) on an ABI 7500 Real-Time PCR System (Thermo Fisher Scientific, USA) according to the manufacturer’s instructions. *β-actin* was used as the internal reference gene for normalization. *β-actin* has been widely used in fish gene-expression studies and has been reported as a relatively stable candidate reference gene in crucian carp [14]. Relative transcript levels were calculated using the 2^−ΔΔCt^ method described by Livak and Schmittgen [15], which assumes comparable amplification efficiencies between the target genes and the reference genes. Amplification specificity was confirmed by the amplification and melting curves.

### 2.5. Immune Challenge

At 28th day post-vaccination, 15 healthy crucian carp were selected randomly from both the vaccinated and control groups and intraperitoneally challenged with 0.2 mL of a virulent *A. veronii* suspension (viable count ≈ 6.5 × 10^6^ CFU/mL). Fish were fasted for 24 h before challenge and for 48 h after challenge. All groups were maintained at 27–29 °C, monitored for 7 days, and daily mortalities were recorded. The relative percent survival (*RPS*) was then calculated according to the following formula:

$$\mathrm{RPS}\left(\%\right)=\left(1-\frac{M_{i}}{M_{c}}\right)\times 100$$

where *Mi* and *Mc* represent the mortality rates of the immunized group and control group, respectively.

### 2.6. Transcriptome Sequencing

#### 2.6.1. Tissue Collection

For each replicate, livers from 10 crucian carp livers were pooled to generate one composite sample for RNA extraction.

#### 2.6.2. Sample Processing and Data Analysis

RNA extraction and quality assessment were performed as described in Section 2.4. After samples passed quality control, eukaryotic mRNA was enriched using mRNA Capture Beads, then mRNA was fragmented via heat treatment in the presence of divalent metal ions. Using fragmented mRNA as the template, first-strand cDNA was synthesized with random hexamer primers, and second-strand cDNA synthesis was subsequently performed. Double-stranded cDNA was purified with DNA Clean Beads, then subjected to end repair, A-tailing, and adaptor ligation. Fragment size selection was performed using DNA Clean Beads, followed by PCR amplification and purification of the amplified products to generate the final libraries. Then, library concentration was initially quantified using a Qubit 3.0 fluorometer (Thermo Fisher Scientific, Waltham, MA, USA), and insert size distribution was assessed with an Agilent 2100 Bioanalyzer. Libraries meeting the expected criteria were then accurately quantified by real-time PCR using an ABI StepOnePlus Real-Time PCR System (Thermo Fisher Scientific, Waltham, MA, USA) to determine the effective library concentration.

#### 2.6.3. Gene Expression Quantification

Based on the read-mapping results, gene expression abundance was quantified using RNA-Seq by Expectation-Maximization (RSEM) (Aegis Biotech Co., Ltd., Guangzhou, China) and normalized as transcripts per million (*TPM*). TPM values were calculated according to the following equation:

$${\mathrm{TPM}}_{i}=\frac{N_{i}/L_{i}}{\sum_{j}\left(N_{j}/L_{j}\right)}\times 10^{6}$$

where $N_{i}$ denotes the number of reads mapped to gene i, $L_{i}$ represents the length (in bases) of gene i, and $\sum_{j}\left(N_{j}/L_{j}\right)$ is the sum of normalized values across all genes.

#### 2.6.4. Differentially Expressed Genes (DEGs) Analysis and Functional Enrichment

Differential expression analysis was performed using edgeR. To control for multiple testing, raw *p* values were adjusted using the Benjamini–Hochberg (BH) procedure, and the resulting adjusted *p* values were reported as false discovery rate (FDR, padj). Unless otherwise stated, genes with padj < 0.05 and |log2 fold change| > 1 were considered differentially expressed. Functional enrichment analyses of DEGs were performed using the GeneDenovo analysis pipeline, based on the Gene Ontology (GO) and Kyoto Encyclopedia of Genes and Genomes (KEGG) databases.

### 2.7. 16S rDNA Sequencing Analysis of Gill Symbiotic Microbiota

After total DNA was extracted from gill samples using a centrifugal column-based genomic DNA extraction kit (Tiangen, Beijing, China)—with three replicates per group—DNA quality, concentration, and integrity were evaluated using the same methods described for RNA. The V3–V4 region of the 16S rRNA gene was PCR-amplified using barcode-tagged primers: *314F* (5′-CCTACGGGNGGCWGCAG-3′) and *806R* (5′-GGACTACHVGGGTATCTAAT-3′) [16]. The purified amplicons were ligated to sequencing adapters, and paired-end sequencing (2 × 250 bp) was performed on an Illumina NovaSeq 6000 platform in PE250 mode. Raw reads were filtered to remove low-quality sequences using USEARCH (v11.0), and high-quality reads were clustered into operational taxonomic units (OTUs). OTU clustering and taxonomic annotation were carried out using UPARSE (https://drive5.com/uparse/, accessed on 25 November 2025) and the RDP Classifier (v2.2), respectively. Krona (v2.6) was used for interactive visualization of taxonomic composition (Interactive metagenomic visualization in a Web browser. BMC bioinformatics). Alpha and beta diversity analyses were conducted using the Vegan package in R. Kyoto Encyclopedia of Genes and Genomes (KEGG) metabolic pathway prediction was performed using PICRUSt2 (version 2.5.3).

### 2.8. Data Analysis

Experimental data are presented as mean ± SD. Prior to analysis, normality and homogeneity of variance were assessed. Statistical significance was evaluated by one-way analysis of variance (ANOVA) using SPSS 14.0 (SPSS Inc., Chicago, IL, USA), followed by the least significant difference (LSD) post hoc test for multiple comparisons. Differences were considered statistically significant at *p* < 0.05. And the correlation analysis after qPCR was performed using Pearson correlation coefficient (*r*), which was calculated according to the following formula [17]:

$$r=\frac{\sum \left(x-\left.\overset{-}{x}\right)\left(y-\overset{-}{y}\right)\right.}{\sqrt{\sum {\left(x-\overset{-}{x}\right)}^{2}\sum {\left(y-\overset{-}{y}\right)}^{2}}}$$

The absolute value of *r* is used to analyze the correlation, the larger the value of |*r*|, the closer the relationship is. Among them, |*r*| < 0.3, means no correlation; 0.3 ≤ |r| < 0.5, means low linear correlation; 0.5 ≤ |*r*| < 0.8, means moderately linear correlation; 0.8 ≤ |*r*| ≤ 1, means highly linear correlation.

## 3. Results

### 3.1. Clinical Signs

**Figure 1:**
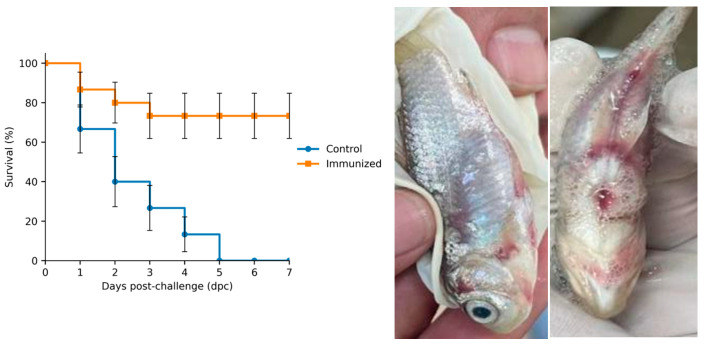
Survival rate of the inactive vaccine (**left**) and clinical signs (**right**) of crucian carp following *A. veronii* challenge. *n* = 15 fish per group; fish were obtained from a single tank per group.

At one day post-injection (dpi) with *A. veronii*, crucian carp in the artificial infection group showed sluggish swimming, reduced appetite, and mortality, with cumulative mortality reaching 100% by 5 dpi. Compared with the control group, infected fish developed exophthalmia and gill hemorrhage, with hyperemia at the bases of the pectoral and ventral fins. Opercular congestion was also observed, and the gill filaments appeared erythematous with ulcerative lesions as described [18]. In addition, scale loss on both sides of the abdomen, abdominal distension, and perianal edema were observed (Figure 1).

### 3.2. RPS of the Inactivated Vaccine

Within 7 days post-challenge (Figure 1), survival was monitored in 15 fish per group (n = 15), with each group maintained in a single tank. The control group exhibited continuous mortality from days 1 to 5. Specifically, there was five deaths on day 1 (survival rate, 66.67%), followed by four additional deaths on day 2 (40.00%), two deaths on day 3 (26.67%), and two deaths on day 4 (13.33%). By day 5, all remaining fish had died (0%). In contrast, the Vac showed mortality early: two deaths on day 1 (86.67%), one death on day 2 (80.00%), and one death on day 3 (73.33%); no further deaths were recorded from days 4 to 7, and survival stabilized at 73.33%. According to the relative percent survival (RPS) calculation, the Vac achieved an RPS of 73.33%.

### 3.3. Expression Profiles of Immune-Related Genes Across Tissues

#### 3.3.1. Liver

**Figure 2:**
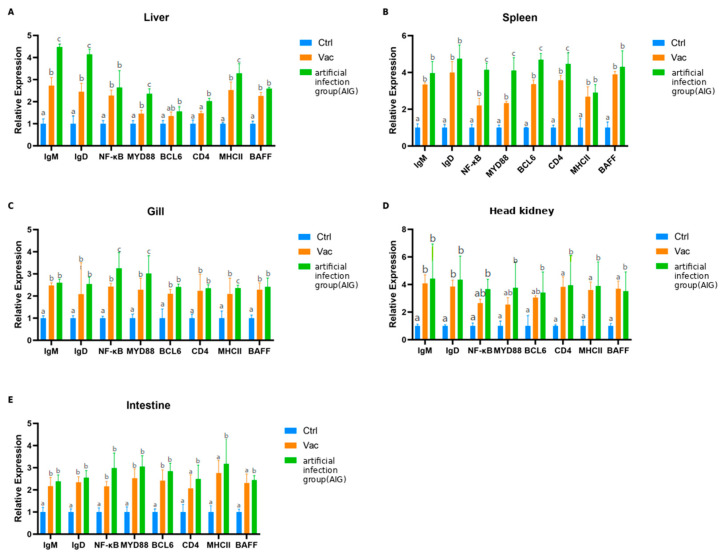
Expression profiles of immune-related genes across tissues. Results were obtained from samples collected at 8 h post-injection. (**A**) liver; (**B**) spleen; (**C**) gill; (**D**) head kidney; (**E**) intestine. Blue, control group; orange, vaccinated group; green, AIG. Relative mRNA expression levels of *IgM*, *IgD*, *BAFF*, *MHC II*, *CD4*, *BCL6*, *MyD88* and *NF-κB* quantified by the 2^−ΔΔCt^ method and normalized to Ctrl (Ctrl = 1). Data are presented as mean ± SD (n = 10). Different letters indicate significant differences among groups (*p* < 0.05).

*IgM* and *IgD* expression profiles were significantly upregulated in livers of both the Vac and AIGs compared with Ctrl (*p* < 0.05), showing a stepwise increase in the order Ctrl < Vac < AIG. *MHC II* and *MyD88* exhibited a similar graded pattern, with the AIG being significantly higher than both Vac and Ctrl (*p* < 0.05). While *NF-κB*, *BAFF* and *BCL6* expression were significantly elevated in Vac and AIG relative to Ctrl (*p* < 0.05), whereas no significant difference were detected between Vac and AIG (*p* > 0.05) (Figure 2).

#### 3.3.2. Spleen

*IgM*, *IgD*, *MHC II*, and *BAFF* expression profiles were upregulated significantly in the spleen of both the Vac and AIGs relative to Ctrl (*p* < 0.05), whereas no significant differences were detected between Vac and AIG (*p* > 0.05). This pattern indicates that vaccination alone elicited a robust splenic transcriptional activation, with challenge tending to further enhance these responses. In contrast, *NF-κB*, *MyD88*, *BCL6*, and *CD4* increased in the order Ctrl < Vac < AIG more clearly, and all pairwise comparisons were significant (*p* < 0.05), suggesting that post-challenge stimulation was associated with stronger transcriptional responses related to innate immune signaling and lymphocyte-associated immune processes (Figure 2).

#### 3.3.3. Head Kidney

*IgM* and *IgD* expression profiles were increased significantly in the head kidney of both the Vac and AIGs compared with Ctrl (*p* < 0.05), whereas no significant difference was observed between Vac and AIG (*p* > 0.05). *NF-κB*, *MyD88* and *BCL6* were significantly higher in AIG than in Ctrl (*p* < 0.05), while differences between Vac and Ctrl, as well as between Vac and AIG, were not significant (*p* > 0.05). In contrast, *CD4*, *MHC II* and *BAFF* were markedly upregulated in the AIG compared with both Ctrl and Vac (*p* < 0.05), with no significant difference between Ctrl and Vac (*p* > 0.05). Collectively, these results indicate that, in the head kidney as a central immune organ, transcriptional responses associated with immune cell activation and antigen presentation are rapidly induced upon AIG (Figure 2).

#### 3.3.4. Intestine

*IgM*, *IgD*, *NF-κB*, *MyD88* and *BCL6* expression profiles were significantly elevated in both the intestine of the Vac and AIGs compared with the Ctrl (*p* < 0.05), whereas no significant differences were detected between Vac and AIG (*p* > 0.05), indicating that vaccination alone was sufficient to induce a robust intestinal immune transcriptional response. In contrast, *CD4*, *MHCII* and *BAFF* were higher significantly in the AIG than in both Ctrl and Vac (*p* < 0.05), with no significant difference between Ctrl and Vac (*p* > 0.05). These results suggest that, relative to the inactivated vaccine, live bacterial exposure more strongly drives intestinal immune cell activation and antigen presentation-associated processes (Figure 2).

#### 3.3.5. Gill

All measured gene expression profiles were significantly higher in the gills of both Vac and AIG than in Ctrl (*p* < 0.05). Notably, *NF-κB*, *MyD88* and *MHCII* were further and significantly increased following AIG (*p* < 0.05), showing a clear stepwise pattern (Ctrl < Vac < AIG), which indicates that MALT reinforce innate signaling and antigen presentation upon pathogen exposure. In addition, *IgM*, *IgD*, *BCL6*, *CD4* and *BAFF* levels were elevated in both Vac and AIG compared with Ctrl, whereas no significant differences were detected between Vac and AIG (*p* > 0.05). These results suggest that intraperitoneal vaccination alone markedly enhances gill immune-related transcription, while the incremental increase after challenge is comparatively limited (Figure 2).

#### 3.3.6. Correlation Analysis

**Figure 3:**
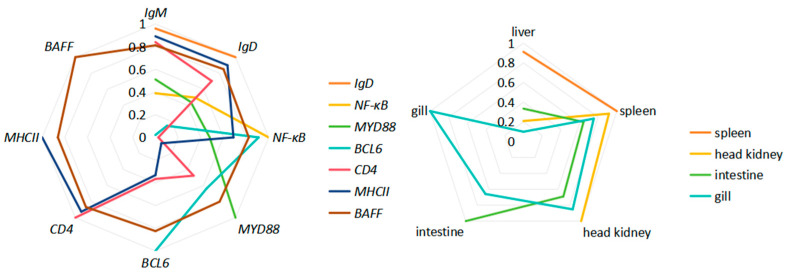
Correlation analysis of *IgM*, *IgD*, *BAFF*, *MHC II*, *CD4*, *BCL6*, *MyD88* and *NF-κB* (**left**) and different organs (**right**) after vaccination and artificial infection.

Based on the measured expression profiles of immune-related genes across different tissues and organs, correlation analysis was performed according to the method described [17]. The results showed that among the immune-related genes, although *IgM* and *IgD* exhibited the highest correlation (*r* = 0.9594), some genes, such as *BCL6* and *NF-κB*, showed correlation coefficients below 0.5 or even below 0.3, indicating very weak correlations. In contrast, *BAFF* showed *r* values greater than 0.8 with all immune-related genes, suggesting that *BAFF* may serve as a representative indicator for evaluating host immune status. Among the different tissues and organs examined, the liver and spleen displayed a high correlation (*r* = 0.9115), which was similar to the findings reported in Reference [17], indicating a strong immunological coordination between these two organs (Figure 3).

### 3.4. DEGs Analysis

**Figure 4:**
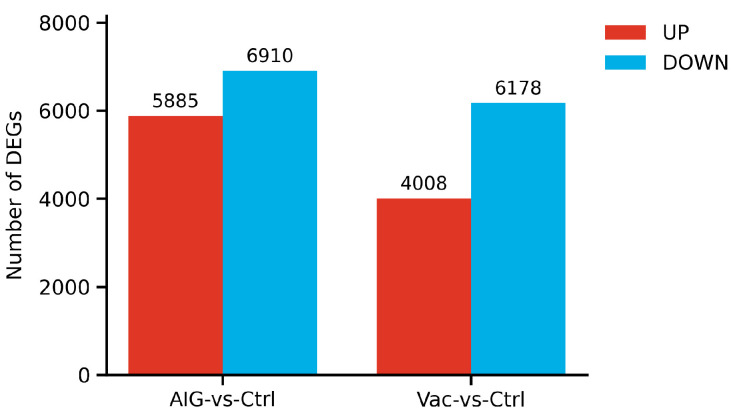
Differences in gene statistical figure.

As shown in Figure 4, in the samples collected at 8 h post-injection, the number of DEGs identified from pairwise comparisons among treatments are summarized in a bar chart, where red and blue bars denote upregulated and downregulated genes, respectively. AIG vs. Ctrl exhibited a substantial DEG repertoire, with 5885 upregulated and 6910 downregulated genes. While Vac vs. Ctrl also exhibited a substantial DEGs repertoire, with 4008 upregulated and 6178 downregulated genes.

### 3.5. GO Functional Enrichment Analysis

**Figure 5:**
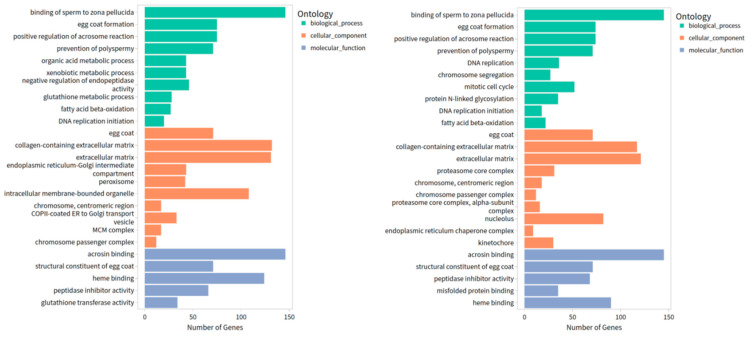
Go terms of AIG vs. ctrl (**left**) and vac vs. ctrl (**right**).

As shown in Figure 5, GO enrichment analyses were performed for AIG vs. Ctrl and Vac vs. Ctrl, covering the three major GO categories: Biological Process, Cellular Component, and Molecular Function. The enriched gene sets largely overlapped between the two contrasts, suggesting broadly comparable immunophysiological responses. Nevertheless, given the differences in antigenic activity—particularly because the vaccine was inactivated—distinct enrichment patterns were also observed for metabolism-related genes.

For AIG, DEGs were predominantly enriched in Biological Process terms associated with cellular and metabolic activities. The most represented terms included cellular process, biological regulation, metabolic process, and localization. Among the Cellular Component category, the term with the highest number of enriched genes was cellular anatomical entity. While among the Molecular Function category, the top enriched terms were binding, catalytic activity, and transcription regulator activity.

In Vac, the most enriched Biological Process terms were biological regulation, cellular process, metabolic process, and localization. Consistent with the AIG, the Cellular Component category was dominated by cellular anatomical entity. Within the Molecular Function category, the top terms included binding, catalytic activity, and molecular function regulator activity.

### 3.6. KEGG Pathway Enrichment Analysis

**Figure 6:**
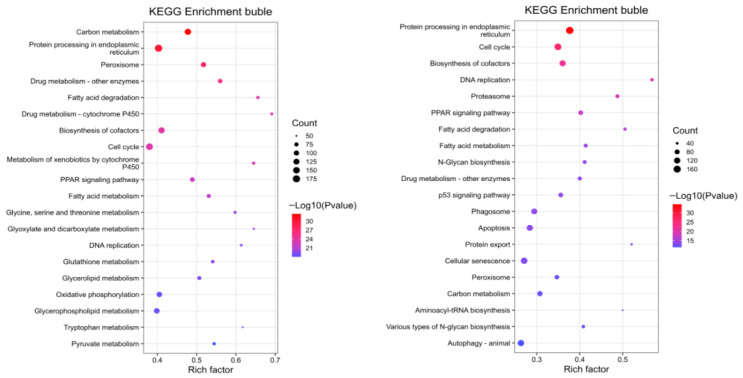
KEGG enrichment bubble plots of DEGs for the AIG (**left**) and Vac (**right**) comparisons. Bubble size indicates the number of enriched DEGs, and bubble color denotes enrichment significance (*p* < 0.05), where q-values represent FDR-adjusted *p*-values.

As shown in Figure 6, 163 pathways were enriched in AIG vs. Ctrl, of which 127 were significant (*p* < 0.05), accounting for 77.91%. The bubble plot presents the top 20 pathways ranked by significance, with enriched gene counts ranging from 50 to 183 and enrichment ratios from 0.380 to 0.691. Among the significantly enriched pathways, carbon metabolism exhibited the highest significance. Protein processing in endoplasmic reticulum contained the largest number of enriched genes, with 183 DEGs. Pathways about xenobiotic biotransformation were also prominently enriched, including drug metabolism—cytochrome P450 and drug metabolism—other enzymes. In addition, multiple lipid and energy metabolism pathways were also overrepresented significantly (*p* < 0.05), including Fatty acid degradation, the *PPAR* signaling pathway, and Glutathione metabolism. These results indicate that AIG-induced transcriptional reprogramming in the liver was primarily centered on metabolic readjustment (carbon, lipid, and energy metabolism) and the maintenance of detoxification and redox homeostasis (cytochrome P450, glutathione, and peroxisome-related processes), accompanied by alterations in cell-cycle and replication-associated programs.

Compared with Ctrl, Vac KEGG enrichment analysis was performed using 3612 DEGs as input. A total of 164 pathways were enriched, among which 131 pathways reached significance (*p* < 0.05), accounting for 79.88%. The bubble plot depicts the top 20 pathways ranked by significance, with Count values ranging from 36 to 171 and Rich factor values from 0.263 to 0.568. Among the significantly enriched pathways, protein processing in endoplasmic reticulum showed the highest significance and also contained the largest number of enriched genes (171 DEGs), followed by cell cycle and biosynthesis of cofactors. Pathways related to proteostasis were particularly prominent in the Vac, including proteasome (59 DEGs) and protein export (38 DEGs). Notably, the canonical immune-related pathway phagosome was significantly enriched, comprising 112 DEGs. In addition, metabolism-associated pathways remained overrepresented, such as the PPAR signaling pathway and fatty acid degradation.

**Figure 7:**
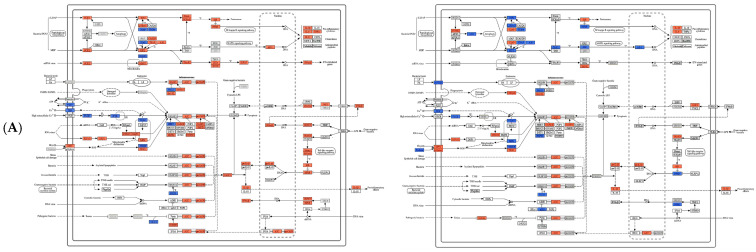

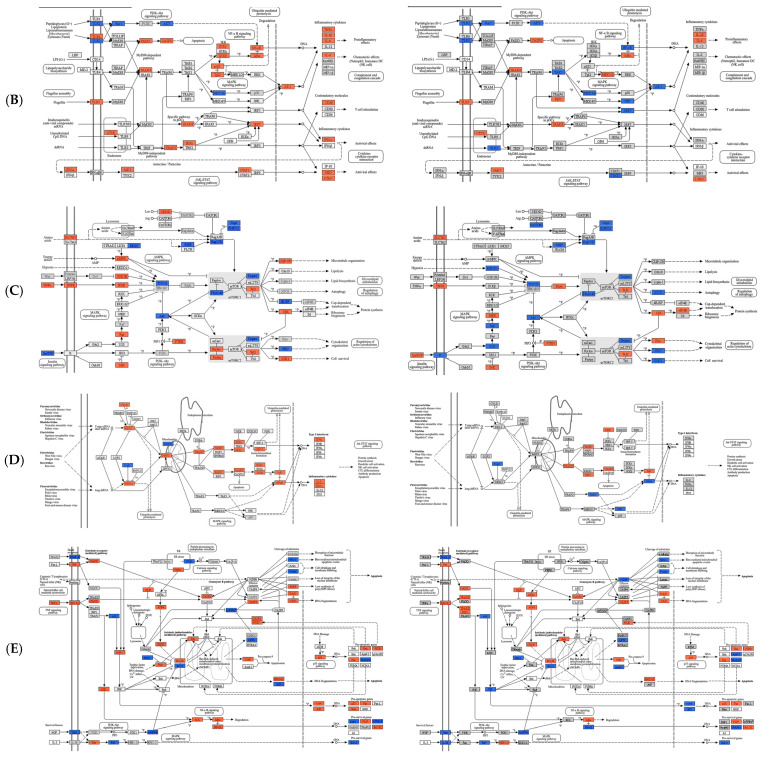
Signaling pathway map in the liver of fish following *A. veronii* AIG (**left**) and vaccination (**right**) (**A**): *NOD-like* receptor signaling pathway; (**B**): *TLR* signaling pathway; (**C**): *mTOR* signaling pathway; (**D**): *RIG-I-like* receptor signaling pathway; (**E**): *Apoptosis* signaling pathway. Red and blue boxes indicate differentially expressed genes (DEGs), with red denoting upregulated expression and blue denoting downregulated expression.

KEGG pathway interrogation highlighted the involvement of multiple inflammation- and immunity-related signaling cascades in the hepatic response, including the *NOD-like* receptor signaling pathway, *TLR* signaling pathway, *RIG-I*-like receptor signaling pathway, *mTOR* signaling pathway, and apoptosis (Table 2). Specifically, 147 DEGs mapped to the *NOD-like* receptor pathway, 79 to the *TLR* pathway, 43 to the *RIG-I-like* receptor pathway, 97 to the *mTOR* pathway, and 148 to the apoptosis pathway (Figure 7). Distinct differences were observed between the AIG and Vac across several pathways. For example, *BCL6*, *MyD88*, and *NF-κB* were upregulated after *A. veronii* infection, whereas in the Vac, *NF-κB* exhibited a downregulated trend in the *TLR* signaling pathway. Collectively, these results indicate that although the overall responses appeared broadly comparable, the DEG landscapes and pathway engagement differed across treatments.

### 3.7. Quality Control of 16S rRNA Sequencing Data

After filtering, 98.94–99.60% of reads were retained, yielding 1.18 × 10^5^–2.93 × 10^5^ clean reads per library (Ctrl: 140,716; Vac: 118,202; AIG: 293,340). Base-level quality was consistently high (Q20: 93.59–97.00%, Q30: 87.79–92.19%) (Table 3). These Q-score metrics indicate low expected base-calling error rates at Q20/Q30 and are commonly used benchmarks for assessing Illumina sequencing quality.

### 3.8. ASV Feature Profiling and Summary

ASV-based profiling (amplicon sequence variant) was summarized across multiple taxonomic levels. ASVs are increasingly preferred over OTUs because they provide solutions and improve comparability across datasets. Across samples, 13 phyla were detected in each library. The number of annotated taxa was higher in Vac and AIG than in Ctrl at finer ranks: at the genus level, Ctrl had 76 genera versus 108 (Vac) and 100 (AIG); at the species level, Ctrl had 81 species versus 117 (Vac) and 108 (AIG) (Table 4).

### 3.9. Alpha-Diversity Analysis

Alpha-diversity analysis further indicated that microbial richness and diversity were generally higher in the Vac and AIGs than in Ctrl at 8 h post-injection. Specifically, Observed species and Chao1 were 191 and 199 in Vac and AIG, respectively, compared with 134 and 135.5 in Ctrl. The Shannon index increased to 3.61–3.69 in Vac and AIG, whereas Ctrl remained at 3.19; Pielou’s evenness likewise rose to 0.69–0.70 in Vac and AIG relative to 0.65 in Ctrl. Meanwhile, Good’s coverage approached saturation (0.9997–1.0000), indicating that sequencing depth captured the vast majority of detectable taxa. Consistently, phylogenetic diversity (PD whole tree) was highest in Vac (15.15), followed by AIG (13.51) and Ctrl (10.34) (Table 5).

### 3.10. Beta-Diversity Analysis

**Figure 8:**
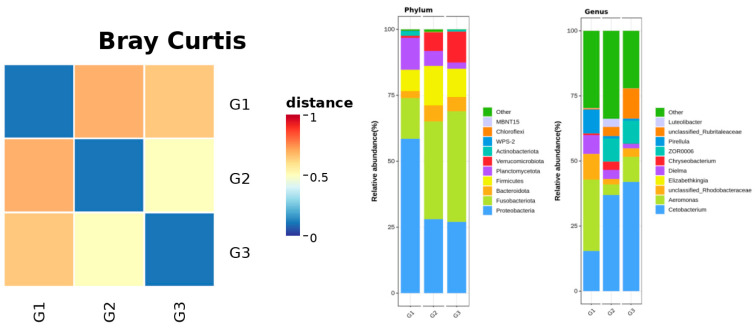
Bray–Curtis-based β-diversity analysis (**Left**); Phylum-level (**Middle**) and Genus-level taxonomic composition analysis (**Right**); G1: AIG; G2: Vac; G3: Ctrl.

Bray–Curtis-based beta-diversity analysis revealed a clear separation in community structure among all groups at 8 h post-injection. The distance heatmap showed that Vac was more similar to Ctrl, whereas AIG exhibited larger dissimilarities relative to both Vac and Ctrl, indicating a pronounced overall shift in microbial community composition following pathogen challenge (Figure 8).

The gill symbiotic microbiota at the phylum level was dominated by Proteobacteria and Fusobacteriota across all groups, yet their relative contributions displayed distinct treatment-associated patterns. AIG were characterized by the highest proportion of Proteobacteria (exceeding 50% of the community), accompanied by a comparatively reduced abundance of Fusobacteriota. In contrast, Vac and Ctrl showed a marked increase in Fusobacteriota, which emerged as a major constituent, with a concomitant decrease in Proteobacteria, indicating greater similarity between the immunized and control profiles and a more pronounced phylum-level compositional shift following pathogen challenge. Beyond these dominant phyla, Firmicutes, Bacteroidota, Planctomycetota, and Verrucomicrobiota were consistently detected, but varied in abundance among treatments: Verrucomicrobiota was generally more abundant in Vac and Ctrl than in AIG, whereas Planctomycetota was relatively enriched in AIG. These results suggested that pathogen challenge was associated with more pronounced alterations in phylum-level community structure, whereas Vac group remained broadly closer to Ctrl in overall composition (Figure 8).

At the genus level (Figure 8), all three groups contained a substantial “Other” fraction, indicating that the gill symbiotic microbiota comprised a diverse set of low-abundance taxa. Among the dominant genera, Cetobacterium was more abundant in Vac and Ctrl, whereas *A.* spp. was relatively enriched in AIG. And several conditionally pathogenic genera (Chryseobacterium and Elizabethkingia) fluctuated in relative abundance across treatments, suggested treatment-specific effects on both core dominant taxa and potential opportunistic members of the gill mucosal community.

## 4. Discussion

### 4.1. Gene Expression Profiles Differed Markedly Between Vaccination and Challenge

Many previous studies have demonstrated that an inactivated *A. veronii* vaccine confers effective protection in crucian carp [1,2]. In the present experiment, the inactivated bacteria cannot replicate in vivo or cause overt infection, but the antigenic components remain sufficient to elicit an immune response [19,20]. Consistent with this, overall transcriptional activation was more pronounced after challenge than after vaccination, particularly for *NF-κB* and *MyD88*. Notably, both genes exhibited a clear stepwise pattern (AIG > Vac > Ctrl) in gills, indicating sustained enhancement of innate antigen recognition in MALT following challenge. This heightened response may further promote the production of inflammatory mediators and subsequently reshape systemic metabolism, as reflected by increased energy and accelerated lipid catabolism [21,22,23]. There were 4907 DEGs identified in spotted sea bass (*Lateolabrax maculatus*) infected with *A. veronii*, mainly associated with cellular redox regulation and protein phosphorylation [24]. In the present study, DEGs were more abundant in AIG than in the vaccine group (5885 vs. 4008 up-regulated; 6910 vs. 6178 down-regulated). Although the top GO terms were broadly comparable between contrasts, their underlying gene compositions differed subtly (Figure 6). Following challenge, the observed transcriptional shifts were mainly associated with genes related to immune-cell functions, particularly those involved in T-cell- and B-cell-related immune processes.

Notably, *BAFF*, *BCL6*, *IgM* and *IgD* in the spleen more frequently showed higher expression in AIG than in Vac, whereas in the head kidney both Vac and AIG tended to be up-regulated, with a comparatively limited additional increase after challenge. This pattern supports the notion that humoral responses, once initiated, undergo a more evident secondary amplification within the spleen [6,8,9]. Interestingly, *BAFF* expression was strongly correlated with all immune-related parameters (Figure 3), previous studies have reported that teleost *BAFF* can be induced across tissues following bacterial infection or antigenic stimulation and can promote *IgM* production and B-cell functionality [24]. In line with these observations, our data suggest that *BAFF*, among all markers, may serve as a particularly informative cytokine indicator for evaluating the magnitude of humoral immune efficacy.

### 4.2. Liver Is an Important Fish Immunometabolic Organ

In teleost fish, the liver is an important immuno-metabolic organ that contributes to metabolic regulation and acute-phase immune responses, and may coordinate with classical immune organs during host defense [25,26]. In crucian carp, hepatic expression of *MyD88*, *NF-κB*, *IgM*, and *IgD* generally showed a stepwise increase (AIG > Vac > Ctrl), consistent with previous reports of bacterial infection in fish [27,28]. In a zebrafish model of *A. hydrophila* infection, liver DEGs were significantly enriched in redox-related terms and metabolic regulatory pathways, including PPAR signaling [29]. Similarly, proteomic studies have shown that *A. hydrophila* infection simultaneously disrupted carbon metabolism and immune signaling, while also affecting the metabolism of xenobiotics by cytochrome *P450* [30]. In the present study, KEGG analysis further revealed that several immunity-related pathways were involved in the hepatic response of challenged fish, including the NLR, TLR, and RIG-I-like receptor signaling pathways, as well as mTOR signaling and apoptosis. Collectively, these findings suggest that the host modulates immune-related gene expression to maintain an appropriate balance between effective antimicrobial defense and the prevention of excessive tissue damage.

Through multiple signaling networks, immune cells can actively reshape metabolism at both the tissue and systemic levels. Although different treatments may yield broadly comparable transcription outputs, the underlying regulatory architecture can differ substantially (Figure 8), reflecting stimulus-specific tuning of cellular energy demands and metabolic substrates [8,31,32]. In human immunology, pathogen associated molecular patterns sensed by innate receptors, such as TLR agonists in macrophages and dendritic cells, as well as antigen receptor signaling via the TCR and BCR in lymphocytes, can rapidly reprogram these cells, redirecting them toward distinct metabolic substrates and pathways [31,33,34]. Among metabolic tissues, adipose-resident immune cells are regarded as particularly prominent contributors to immunometabolic regulation [35], whereas systemic lipid homeostasis is largely orchestrated by the liver [36].

In the liver, several additional immune indices, including *MHC II*, *CD4*, *IgM*, *IgD*, *BAFF*, and *BCL6*, showed a clear stepwise increase (Ctrl < Vac < AIG). This pattern indicates that, during pathogen challenge, the liver may serve functions beyond metabolic regulation and participate in systemic immunity through antigen presentation, T-cell-associated activity, and B-cell-mediated responses, with cytokine support likely involved [8,37]. This interpretation is supported by the study that immersion vaccination of rainbow trout with *Yersinia ruckeri* significantly elevated *IgM* transcripts in the head kidney, whereas the posterior kidney did not exhibit the expected increase, owing to the greater abundance of *IgM*-secreting plasma cells in the head kidney [38]. Similarly, in the present study, hepatic enrichment of *PRR*-related pathways, together with activation of *mTOR* and apoptosis signaling and increased transcription of *BAFF* and immunoglobulins, further supports an immunoregulatory role of liver during infection.

Notably, we found that spleen showed stronger coordinated responsiveness during challenge, pointing to a more prominent amplification role with liver. Following immersion with an inactivated *Vibrio harveyi* vaccine in crucian carp, grouper, and related species, organ-specific *IgM* mRNA levels were reported to fluctuate over time, with the hindgut, liver, and spleen following similar temporal trajectories, leading to the proposal of a “hindgut–liver–spleen” axis [17]. In the present study, a strong correlation was observed between the liver and spleen, whereas the correlation with the intestine was relatively weak, which differed from previous reports. Therefore, we can infer that since the liver is so crucial in the infection immunity process and even collaborates with the spleen’s immune function, in the process of fish farming production management, it is necessary to enhance the health maintenance of the liver, improve material metabolism, accelerate growth, and also boost immunity to ensure production safety.

### 4.3. The Gill Symbiotic Microbiota Is Also Affected by Vaccination and Challenge

Fish gills mucus layer and symbiotic microbiota serve as a physical barrier and a chemically active interface, thereby constituting the first line of defense [39,40,41]. Pathogen challenge is often accompanied by epithelial damage, alterations to the mucus barrier, and local inflammation [42]. And this can destabilize community structure and facilitate the expansion of opportunistic taxa [43]. Consistent with this concept, studies in grass carp infected with *Ichthyophthirius multifiliis* have reported marked shifts in gill symbiotic microbiota, suggesting that disrupted ecological balance may favor opportunistic bacterial proliferation [44]. Similar observations have been made in goldfish infection models, where gill pathological lesions were accompanied by changes in gill-associated β-diversity and fluctuations in opportunistic taxa, supporting the notion that parasitic infection can drive mucosal microbiome reorganization [45].

Phylum-level profiling corroborated this pattern: all groups were dominated by Proteobacteria and Fusobacteriota, yet AIG was characterized by an increased relative abundance of Proteobacteria and a concomitant reduction in Fusobacteriota; in contrast, Vac and Ctrl displayed higher Fusobacteriota and lower Proteobacteria, resulting in closer overall compositions between the two groups. In line with broader evidence from other teleost mucosal microbiome studies, infection or severe stress frequently induces pronounced perturbations in gill microbial communities, often including Proteobacteria enrichment as an indicator of compromised community homeostasis, whereas vaccination tends to elicit modest remodeling or the maintenance of stability [44,45,46].

## 5. Conclusions

By contrasting intraperitoneal vaccination with an inactivated *A. veronii* vaccine against acute challenge, we show that artificial infection elicited a markedly stronger host response, with *BAFF* transcription across organs emerging as a robust sentinel readout for its *r* values which all exceed 0.8. As an important immunometabolic organ, the liver mounted a coordinated response characterized by antioxidant defense, detoxification, and metabolic reprogramming, and appeared to act in concert with the spleen to support protective immunity. Of course, more molecular detail about liver–spleen coordination should be presented in the future. For couples host immune readouts with gill mucosal microbiota dynamics, gill should be recognized as a frontline barrier where immune activation and microbial ecology jointly shape disease trajectories, and its microecology of symbiotic bacteria was affected by vaccination or challenge reactions, which in turn affects the health of the gills or the organism itself.

## Figures and Tables

**Table 1:** Real-time quantitative PCR (qPCR) primer sequences.

Gene Name	Primer Sequences (Forward/Reverse) (5′–3′)	Accession No.
*β-actin*	F: CAAGATGATGGTGTGCCAAGTG R: TCTGTCTCCGGCACGAAGTA	LOC132158096
*IgM*	F: GTGGAACTTGATGCCCCAAT R: CATCAGCAAGCCAAGACACAA	LOC109106335
*IgD*	F: TGGGTCCATCCCTCAGTAGT R: GTTGTTTGGGGTCTCGGGTT	LOC132139794
*NF-κB-* *α*	F: GCCACTAAATCCACCACATC R: AACCCAAGCAGTTCACATACA	LOC132110750
*MyD88*	F: CTATGAGGCGATTCCAGTAACA R: CCAGTCTGCTGCCACCG	LOC132123839
*BCL6*	F: CAACAACATCATCAACAGCAGAAC R: CTCATACAGAGCGAAGAGTGGC	LOC132151890
*CD4*	F: ACAACTGCCAATCAGACGGAG R: TGCAGCGGATGTGTTCACTTA	LOC132157733
*MHCII*	F: GGACATCAGACCCTGGACCAA R: ACACCGAGCAGACCGACAGT	LOC113046967
*BAFF*	F: CTGTAAGGAGGATGTTTTGAGTTG R: GGAAGAGGAGGTGATGGTAGC	LOC113108918

**Table 2:** Pathway analysis of DEGs in the crucian carp liver.

Pathway	DEGs	*p*-Value	Q-Value
*NOD-like* receptor signaling pathway	147	4.492887 × 10^−18^	9.888652 × 10^−18^
*TLR* signaling pathway	79	3.010973 × 10^−7^	2.024924 × 10^−7^
*RIG-I-like* receptor signaling pathway	43	0.0087575666	0.0039630896
*mTOR* signaling pathway	97	0.0222010195	0.0098623553
*Apoptosis* signaling pathway	148	1.173637 × 10^−17^	2.367864 × 10^−17^

**Table 3:** Quality assessment of 16S rRNA amplicon sequencing data.

Sample	Raw Reads	Clean Reads (%)	Raw Bases (bp)	Clean Bases (bp)	Q20 (%)	Q30 (%)	GC (%)
AIG	293,340	98.94	66,756,424	3,8867,086	93.59	87.79	52.56
Vac	118,202	99.59	26,825,936	23,186,472	96.89	92.17	50.67
Ctrl	140,716	99.60	31,937,717	28,116,102	97.00	92.19	50.80

**Table 4:** Summary of ASV features.

Sample	Phylum	Class	Order	Family	Genus	Species
AIG	13	16	40	61	100	108
Vac	13	20	52	77	108	117
Ctrl	13	15	38	54	76	81

**Table 5:** Alpha-diversity analysis.

Index	Observed Species	Chao1	Shannon	Simpson	Pielou	Coverage	PD Whole Tree
AIG	199	199	3.69	0.94	0.7	1	13.51
Vac	191	191.18	3.61	0.9	0.69	0.9999	15.15
Ctrl	134	135.5	3.19	0.89	0.65	0.9997	10.34

## Data Availability

Data will be made available on request.
